# Top1p targeting by Fob1p at the ribosomal Replication Fork Barrier does not account for camptothecin sensitivity in *Saccharomyces cerevisiae* cells

**DOI:** 10.17912/micropub.biology.000514

**Published:** 2022-01-19

**Authors:** Pardis Pourali, Philippe Pasero, Benjamin Pardo

**Affiliations:** 1 Institut de Génétique Humaine, Université de Montpellier-CNRS, Montpellier, France

## Abstract

Camptothecin (CPT) is a specific inhibitor of the DNA topoisomerase I (Top1p), currently used in cancer therapy, which induces DNA damage and cell death. Top1p is highly active at the repeated ribosomal DNA *locus* (rDNA) to relax DNA supercoiling caused by elevated transcription and replication occurring in opposite directions. Fob1p interacts with, and stabilizes, Top1p at the rDNA Replication Fork Barrier (rRFB), where replication and transcription converge. Here, we have investigated if the absence of Fob1p and the consequent loss of Top1p specific targeting to the rRFB impact the sensitivity and the cell cycle progression of wild-type cells to CPT. We have also investigated the consequences of the absence of Fob1p in *rad52∆* mutants, which are affected in the repair of CPT-induced DNA damage by homologous recombination. The results show that CPT sensitivity and the global cell cycle progression in cells exposed to CPT is not changed in the absence of Fob1p. Moreover, we have observed in *fob1∆* cells treated with CPT that the homologous recombination factor Rad52p still congregates in the shape of foci in the nucleolus, which hosts the rDNA. This suggests that, in the absence of Fob1p, Top1p is still recruited to the rDNA, presumably at sequences other than the rRFB, and its inhibition by CPT leads to recombination events.

**Figure 1. Consequences of FOB1 deletion on growth, cell cycle progression and Rad52p foci formation in response to CPT:**
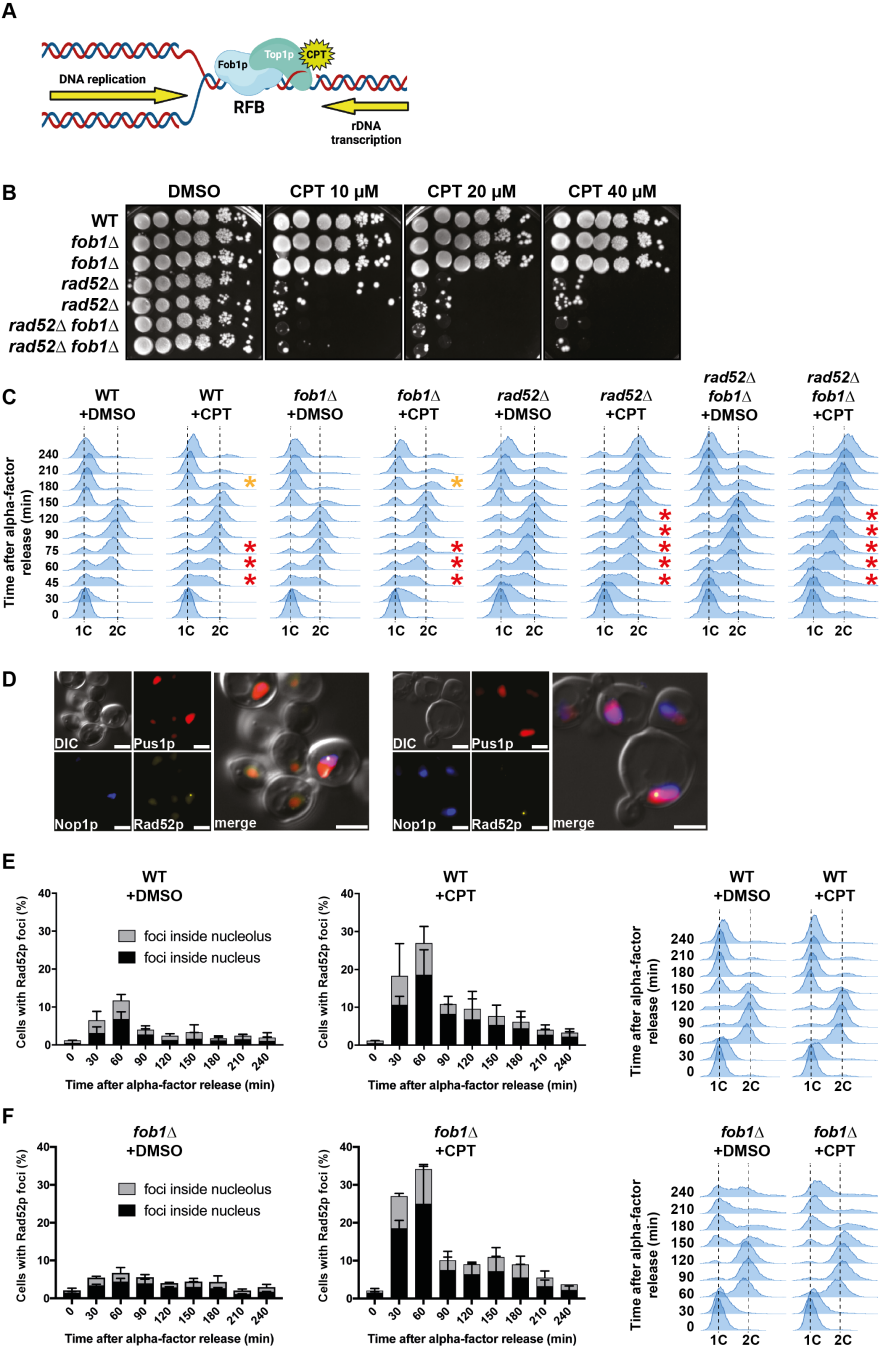
**(A)** Schematic representation of the ribosomal Replication Fork Barrier (rRFB), where DNA replication and rDNA transcription converge. Fob1 sits at the rRFB and recruits Top1p. Top1p cleavage complexes (Top1ccs) can be stabilized by camptothecin (CPT). **(B)** Tenfold serial dilutions of cells of the indicated genotypes were grown on rich YPAD medium plates containing DMSO (control) or CPT at the indicated concentrations. Growth plates were incubated for 2 days at 30°C. **(C)** Cell cycle progression analysis. Exponentially growing cell cultures were synchronized in G_1_ using α-factor and released into S phase in the presence of DMSO (control) or 100 µM CPT. 90 minutes after release, α-factor was again added, preventing the cells to progress through the next cell cycle. Cell samples were harvested at the indicated time points to evaluate DNA contents by flow cytometry. The red stars indicate the S-phase delay of CPT-treated cells compared to their DMSO controls and the orange stars indicate when most cells reach the following G_1_ phase. **(D-F)** Rad52p foci formation analysis. **(D**) Two representative examples of Rad52p-YFP foci (yellow) being formed in the nucleolus (marked by Nop1p-CFP, blue) or in the nucleus (marked by mCherry-Pus1p, red) as visualized by fluorescence microscopy. DIC: differential interference contrast. Scale bar = 3µm. **(E, F)** Cell culture was performed similarly as described in **(C)** and samples were harvested at the indicated time points to evaluate DNA contents by flow cytometry and to score the percentage of cells containing Rad52p foci in the presence of DMSO (control) or 100 µM CPT by fluorescence microscopy. Mean values +/- standard error of the mean (SEM) are indicated.

## Description

DNA topoisomerases play a vital role in solving topological constrains during DNA replication and transcription (Pommier *et al.* 2016). Top1p is a DNA topoisomerase that relaxes DNA supercoiling by nicking the DNA, creating a covalent bond between the enzyme and the 3’ end of the DNA, called Top1p cleavage complex (Top1cc). Once the DNA is relaxed, Top1p religates the break by reversing its covalent binding. Top1p activity can be inhibited by drugs such as camptothecin (CPT), whose derivatives (topotecan, irinotecan) are largely used in cancer therapy. CPT binds to the Top1cc and delay the religation reaction, thus blocking Top1p on DNA (**Figure 1A**) (Pommier *et al.* 2006). It has been shown that treatment of cells with CPT induces DNA double-strand breaks (DSBs) specifically during DNA replication. One model proposes that the DNA nick is converted into a DSB by the passage of the replication fork, which separates the parental duplex DNA (Strumberg *et al.* 2000). Because CPT prevents Top1p ability to remove topological stress (Koster *et al.* 2007), another model proposed that the accumulation of topological constrains would block replication fork advance and induce DSB formation as a consequence of fork cleavage by the MUS81 nuclease (Regairaz *et al.* 2011). These DSBs must be repaired, otherwise mutations could arise and affect genome stability. DSBs are mainly repaired by Homologous Recombination (HR) during the S and G_2_ phases of the cell cycle, the Rad52p protein being a key player in this pathway in yeast *Saccharomyces cerevisiae* (Pardo *et al.* 2009). Rad52p proteins relocalize into discrete sub nuclear foci upon DSB formation (Lisby *et al.* 2003). In response to CPT treatment, yeast cells accumulate Rad52p foci both in the nucleus and the nucleolus (Stuckey *et al.* 2015). These results suggest that CPT-induced DSBs arise in the ribosomal DNA (rDNA) genes, which reside in the nucleolus. These genes are organized on the chromosome XII in a single cluster of 150-200 tandem repeats (Kobayashi 2011). Each rDNA repeat includes a Replication Fork Barrier (rRFB) bound by Fob1p, whose function is to avoid collisions between replication and transcription machineries by stalling the movement of replication forks in only one direction (Kobayashi and Horiuchi 1996). In the rDNA, it has been shown that Top1ccs accumulate naturally and specifically at the rRFB, and that this accumulation is completely lost in the absence of Fob1p (Krawczyk *et al.* 2014). DSBs have also been detected at the rRFB and are dependent on Top1p and Fob1p. These DSB signals were increased upon the inhibition of Top1p by CPT (Krawczyk *et al.* 2014), suggesting that the accumulation of Fob1p-dependent Top1ccs at the rRFB leads to DSB formation. Since the rDNA represents about 10% of the genome size of *Saccharomyces cerevisiae* and these DSBs could be detrimental for cell survival, we thus investigated if the absence of Fob1p could improve the resistance of yeast cells to CPT.

In order to test if *FOB1* deletion could improve cell resistance to CPT, we combined the *rad52∆* mutation, which confers hypersensitivity to CPT, to the *fob1Δ* mutation. *fob1∆* cells did not show sensitivity to CPT at all tested concentrations (**Figure 1B**). Moreover, the hypersensitivity of *rad52Δ* cells was not alleviated by *fob1Δ* (**Figure 1B**). These results suggest that the presence of Fob1p and the consequent stabilization of Top1ccs at the rRFB is not the main cause of cell death in the absence of Rad52p when exposed to CPT.

We then analyzed the progression of cells by flow cytometry during a single cell cycle in response to CPT exposure. After the synchronization in G_1_ (1C DNA content), cells were incubated with either DMSO (control) or CPT in a minimal medium increasing cell permeability to CPT (Pardo *et al.*, 2020) and released into S phase. WT cells treated with DMSO entered the S phase at 30 minutes and reached the G_2_ phase (2C DNA content) at 90 minutes. At 150 minutes, cells finished mitosis and progressed to the following G_1_, where they remained arrested by a second addition of the synchronizing agent α-factor (**Figure 1C**). WT cells exposed to CPT progressed slower through S phase and reached the G_2_ phase 30 minutes later than control cells. Consequently, these cells reached the following G_1_ with a 30 minutes delay (**Figure 1C**). Cell cycle progression of *fob1∆* cells was comparable to WT, either incubated with DMSO or CPT, respectively. On the contrary, *rad52∆* cells exposed to CPT showed an increased S-phase delay compared to WT (**Figure 1C**), consistent with a previous observation (Pardo *et al.* 2020). Once they reached the G_2_ phase 150 minutes after the release from the G_1_ synchronization, they remained blocked in G_2_/M until the end of the time course experiment, at 240 minutes (**Figure 1C**). These results confirm that Rad52p is important for cells to progress through S phase and complete mitosis when they are exposed to CPT (Pardo *et al.* 2020). *FOB1* gene deletion did not change the cell cycle progression of *rad52∆* cells, whether treated with DMSO or with CPT (**Figure 1C**). These data indicate that the absence of Fob1p does not impact the global cell cycle progression in cells exposed to CPT.

Finally, we used a Rad52 protein tagged with YFP to follow by fluorescence microscopy the formation of Rad52p-YFP foci in response to CPT exposure in WT and *fob1∆* cells. After G_1 _synchronization, cells were released into S phase with DMSO/CPT. In WT cells treated with DMSO, Rad52p foci formed specifically during S-phase (30-60 min after release) and decreased until mitosis completion (**Figure 1E**). The foci were localized both in the nucleolus (marked by Nop1p-CFP) and in the remaining of the nucleus (marked by mCherry-Pus1p) (**Figure 1D-E**). The percentage of WT cells containing Rad52p-YFP foci increased about 3-fold in response to CPT and followed the same kinetics as control cells (**Figure 1E**). *fob1∆* cells showed similar results (**Figure 1F**), suggesting that DNA lesions, presumably DSBs, still form in the rDNA in the absence of Fob1p.

Altogether, these results show that *FOB1* deletion does not suppress the phenotypes induced by CPT exposure in WT or *rad52∆* cells. The fact that Rad52p foci formation increased upon CPT exposure (**Figure 1E**) suggests that Rad52p foci are related to Top1ccs. Rad52p foci still formed in the nucleolus in *fob1∆* cells exposed to CPT (**Figure 1F**), suggesting that Top1ccs can still accumulate in the rDNA, likely at sequences other than the rRFB, in the absence of Fob1p. This is consistent with the low amount of Top1ccs detected in the *35S* gene, located upstream of the rRFB, in the presence or absence of Fob1p without CPT treatment (Krawczyk *et al.* 2014). Even if present in a lower amount, these Top1ccs further stabilized by CPT do not impact the cell cycle progression, nor the cell viability in *fob1∆* cells (**Figure 1B-C**). One explanation could be that they do not give rise to DSBs, which are lethal DNA lesions. Indeed, the DNA fragments detected in the rDNA and interpreted as being the consequence of DSBs (Fritsch *et al.* 2010; Krawczyk *et al.* 2014; Sasaki and Kobayashi 2017) could also correspond to the reversal of replication forks (Kara *et al.* 2021), an event in which the fork goes backward by annealing the newly-synthetized DNA strands together. This hypothesis is supported by the lack of “DSB” signals accumulation at the rRFB in the absence of DSB repair factors (Fritsch *et al.* 2010; Sasaki and Kobayashi 2017; Kara *et al.* 2021) and by the fact that fork reversal events are increased in cells exposed to CPT (Ray Chaudhuri *et al.* 2012; Menin *et al.* 2018). Then, Rad52p foci formation could originate from recombination events initiated from the tip of reversed forks instead of DSBs.

## Methods

***Sensitivity assay***. Yeast cells freshly grown on rich YPAD plates overnight at 30°C were resuspended in water and cell concentration was measured with a CASY flow cytometry apparatus. Cell concentration was normalized to 2×10^8^ cells/ml. Tenfold serial dilutions we done and 5µl drops of each dilution were deposited on solid rich YPAD medium containing DMSO or CPT at different concentrations. Image were taken after 2 days of growth at 30°C. The experiment was repeated three times.

***Flow cytometry***. Exponentially growing cells at 7×10^6^ cells/ml were synchronized in G_1_ by the addition of α-factor at 1 µg/ml for 2 hours at 30°C in rich YPAD medium. After cell synchronization in G_1_, cells were pelleted and resuspended in minimal MPD +SDS medium (0.17% yeast nitrogen base, 0.1% L-Proline, 2% glucose and 0.003% SDS) and incubated for 1h at 30°C in the presence of DMSO (control) or 100 µM CPT and α-factor at 1 µg/ml. Release into S phase was done by removing the α-factor by filtration and resuspending the cells in minimal MPD +SDS medium with DMSO or CPT. α-factor was again added 90 minutes after release. At the indicated time points, cells were recovered and fixed in ethanol 70%, permeabilized in Na-citrate 50 mM buffer, incubated with RNase A 50 µg/ml for 2 hours at 50°C and incubated with proteinase K (PK) 230 µg/ml for 1 hour at 50°C. DNA was stained with propidium iodide at 4 µg/ml for 2 hours at room temperature in the dark. DNA fluorescence was measured using Miltenyi Biotec MACSQuant analyzer and analyzed using FlowJo software. The experiment was repeated twice.

***Microscopy.*** Exponentially growing cells at 7×10^6^ cells/ml cultured in minimal SC medium without leucine were pelleted and resuspended in rich YPAD medium for synchronization in G_1_ by the addition of α-factor at 8µg/ml for 2.5 hours at 25°C. α-factor was removed by filtration and cells were released into S phase in rich YPAD medium containing DMSO (control) or 100 µM CPT. α-factor was again added 90 minutes after release. Cells were collected at the indicated time points and pictures of living cells were captured by fluorescence microscopy (×67) using a Zeiss Axioimager and ZEN software. Images were analyzed using ImageJ software and results were plotted using GraphPad Prism software. The experiment was repeated three times.

## Reagents

Yeast strains used in this study come from the W303 background corrected for the *rad5-535* mutation. Mutant strains were generated by PCR-mediated deletion and genetic crossing using standard methods. The strains used in **Figure 1B-C** (WT PP3241, *fob1∆NAT* PP3277 PP278, *rad52∆klLEU2* PP3279 PP3280, *rad52* *∆klLEU2 fob1∆NAT* PP3281 PP3282) contain the *bar1∆* mutation (without any marker) to facilitate the cell cycle synchronization in G_1_ phase. The strains used for microscopy in **Figure 1D-F** (WT PP4669 PP4670 and *fob1∆NAT* PP4672 PP4673) contain wild-type *BAR1* gene, *RAD52-YFP* and *mCherry-PUS1::URA3* integrated at their endogenous loci and the monocopy centromeric plasmid *NOP1-CFP::LEU2*. Alpha-factor was bought from BIOTEM company (custom synthesis). CPT stock solution was made from (S)-(+)-camptothecin (SIGMA C9911) dissolved in DMSO (SIGMA D8418) at 15 mM.
